# Effects of chalazion and its treatments on the meibomian glands: a nonrandomized, prospective observation clinical study

**DOI:** 10.1186/s12886-020-01557-z

**Published:** 2020-07-11

**Authors:** Junping Li, Dongping Li, Na Zhou, Mengying Qi, Yanzhu Luo, Yuhong Wang

**Affiliations:** 1 Aier Eye Hospital (East of Chendu), Chendu, China; 2 Hankou Aier Eye Hospital, Wuhan, 430021 China; 3 Aier School of Ophthalmology, Central South University, Changsha, China

**Keywords:** Chalazion, Meibography, Meibomian gland, MGD

## Abstract

**Background:**

To observe the effects of chalazion and its treatments on meibomian gland function and morphology in the chalazion area.

**Methods:**

This nonrandomized, prospective observational clinical study included 58 patients (67 eyelids) who were cured of chalazion, including 23 patients (23 eyelids) treated with a conservative method and 35 patients (44 eyelids) treated with surgery. Infrared meibomian gland photography combined with image analysis by ImageJ software was used to measure the chalazion area proportion. Slit-lamp microscopy was employed to evaluate meibomian gland function, and a confocal microscope was used to observe meibomian gland acinar morphology before treatment and 1 month after complete chalazion resolution.

**Results:**

At 1 month after chalazion resolution, the original chalazion area showed meibomian gland loss according to infrared meibomian gland photography in both groups. In patients who received conservative treatment, the meibomian gland function parameters before treatment were 0.74 ± 0.75, 0.48 ± 0.67, and 1.22 ± 0.60, respectively. One month after chalazion resolution, the parameters were 0.35 ± 0.49, 0.17 ± 0.49, and 0.91 ± 0.60, respectively; there was significant difference (*P* < 0.05). The proportion of the chalazion area before treatment was 14.90 (11.03, 25.3), and the proportion of meibomian gland loss at 1 month after chalazion resolution was 14.64 (10.33, 25.77); there was no significant difference (*P* > 0.05). In patients who underwent surgery, the meibomian gland function parameters before surgery were 0.93 ± 0.87, 1.07 ± 0.70, and 1.59 ± 0.76, respectively, and at 1 month after chalazion resolution, they were 0.93 ± 0.82, 0.95 ± 0.75, and 1.52 ± 0.70, respectively; there was no significant difference (*P* > 0.05). The proportion of the chalazion area before surgery was 14.90 (12.04, 21.6), and the proportion of meibomian gland loss at 1 month after chalazion resolution was 14.84 (11.31, 21.81); there was no significant difference (*P* > 0.05). The acinar structure could not be observed clearly in the meibomian gland loss area in most patients.

**Conclusions:**

Chalazion causes meibomian gland loss, and the range of meibomian gland loss is not related to the treatment method but to the range of chalazion itself. A hot compress as part of conservative treatment can improve meibomian gland function at the site of chalazion in the short term.

## Background

Chalazia refers to chronic granulomatous inflammation that occurs in one or several glands in the upper or lower eyelids [1] and is one of the most common eyelid diseases affecting individuals of all ages, including children [2]. As a special eyelid disease, chalazion is closely related to the meibomian gland, and the meibomian gland is responsible for secreting meibum, which can reduce evaporation of the tear film, facilitate lubrication of the ocular surface, and provide a smooth optical surface [3]. Although chalazion is a local lesion of the meibomian gland, previous studies have reported that patients with a history of chalazion, especially recurrent chalazion or multiple chalazion, were more likely to suffer from meibomian gland dysfunction (MGD) and blepharokeratoconjunctivitis (BKC) [4, 5]. Therefore, focusing on the effects of chalazion on the function and morphology of local meibomian glands rather than only on treating chalazion is important, especially for patients with preexisting abnormal meibomian gland function and morphology. However, most prior studies on chalazion only compared the effects of different treatments [6–8]. Therefore, the primary objective of this study was to observe changes in meibomian gland function and morphology in the chalazion area using different methods before and after treatment.

## Methods

### Subjects

From August 2017 to August 2018, 71 patients (82 eyelids) who were cured of chalazion were enrolled in this study conducted at the Hankou Aier Eye Hospital, including 28 patients (28 eyelids) who received conservative treatment and 43 patients (54 eyelids) who underwent surgery. The inclusion criteria were as follows: (a) patients with primary chalazion and a single chalazion on one eyelid; (b) patients who were able to cooperate during examinations and received local anesthesia during surgery; and (c) patients without any other previous treatment. The exclusion criteria were as follows: (i) patients who accepted both conservative treatment and surgery during the study (patients for whom conservative treatment failed who then accepted surgery); (ii) patients with a local anesthetic drug allergy history; and (iii) patients with a concurrent eyelid infection combined with pain (hordeolum, cellulitis or conjunctivitis).

Patients were treated with conservative treatment when either of the following criteria were met: (a) the duration of the chalazion was less than 2 months from the time of the patient’s complaint. (b) The chalazion had a horizontal width of less than 5 mm. Given the difficulty of assessing the total volume of each chalazion, the horizontal width of each chalazion was used to represent this value. The measurement method consisted of directly measuring the horizontal width with an ophthalmic surgical caliper under a slit lamp microscope. (c) The patient refused surgery. Otherwise, patients were treated with surgery (incision and curettage). Patients were considered cured when the chalazion completely resolved.

Because no clear standard for chalazion treatment selection is currently available, the treatment strategy depended on clinical experience and past studies [7–9].

### Ethics statement

This study was approved by the Ethics Committee of Hankou Aier Eye Hospital and followed the tenets of the Declaration of Helsinki. Written informed consent was obtained from all patients.

### Examinations

Each patient completed two parts of the clinical examinations before treatment and at 1 month after complete chalazion resolution: a meibomian gland function assessment and meibomian gland morphology analysis of the chalazion area. All examinations were conducted by the same observer during the whole course of the study.

### Meibomian gland function assessment in the chalazion area

The meibomian gland function evaluation addressed the meibomian gland orifice, meibum quality, and meibomian gland expressibility. All examinations targeted one or several meibomian glands where the chalazion was located. The scores for these examinations were determined according to the 2011 International Workshop on MGD [10]. However, because the number of glands invaded by chalazion differed for each patient, the average score was used in this study (the total scores/the number of glands).

### Meibomian gland orifice

The meibomian gland orifice condition was assessed on a scale of 0 to 3: 0, normal; 1, thin blockage; 2, blockage in the orifices; and 3, severe orifice blockage.

### Meibum quality

Each meibomian gland where the chalazion was located was assessed for quality on a scale of 0 to 3: 0, clear; 1, cloudy; 2, cloudy with debris (granular); and 3, thick, similar to toothpaste.

### Meibomian gland expressibility

Expressibility in the glands where the chalazion was located was assessed on a scale of 0 to 3. Scores were assigned according to the secretion capacity percentage (the number of glands with secretory ability in the corresponding area of chalazion/the total number of glands in the corresponding area) based on the International MGD Working Group Standard: 0, all glands; 1, 60–80% of glands; 2, 20–40% of glands; and 3, no glands.

### Meibomian gland morphology in the chalazion area

#### Infrared meibomian gland photography

**Fig. 1:**
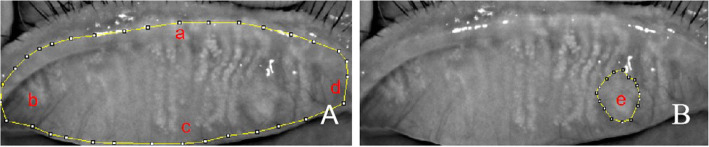
The method of calculating the proportion of chalazion area in the image of the infrared meibomian gland is shown (**a** shows the end of the gland at the upper margin of the iliac crest, **b** shows be the most visible tarsal conjunctiva of the everted lid, **c** shows the gland at the iliac crest, **d** shows the nasal border was defined as the tear punctum, **e** shows the chalazion area). **a** shows the whole area of the tarsal plate, and **b** shows the chalazion area

After the eyelids affected by chalazion were everted, a morphological picture was taken by a noncontact infrared meibography system with the Oculus Keratograph (Oculus GmbH, Wetzlar, Germany) as described previously. ImageJ software was used to calculate the area of the chalazion and the whole area of the tarsal plate where the chalazion was located. The proportion of the chalazion area = the chalazion area/the whole area of the tarsal plate (Fig. 1) [11]. The whole area of the tarsal plate was also used to calculate the area at 1 month after complete chalazion resolution; that is, the denominator was unchanged.

#### In vivo laser scanning confocal microscopy

All patients were examined by in vivo LSCM (HRT III Corneal Rostock Module; Heidelberg Engineering GmbH, Heidelberg, Germany) as described previously [12]. Before each examination, one drop of 0.4% oxybuprocaine eye solution (Santen, Osaka, Japan) was instilled into the conjunctival fornix. After the eyelid where the chalazion was located was everted, the center of the Tomo-cap was applanated onto the palpebral conjunctiva of the chalazion area, the chalazion area was scanned first, followed by the non-chalazion area nearby. The two-dimensional image size was 384 × 384 pixels, with a 400x400μm field of view.

#### Treatments

The conservative treatment method consisted of the following practices: A hot eye mask (Shandong Zhushi Pharmaceutical Group Co., Ltd.) was applied for 10 min in the morning and at night, one drop of 0.5% levofloxacin eye solution was applied four times a day, and ofloxacin eye ointment was applied before bed. The local antibiotics were used for 4 weeks.

The surgical method consisted of the following procedures: 1) The chalazion area was infiltrated with approximately 1 ml of 2% lidocaine, and after local anesthesia infiltration, the eyelid was everted using a chalazion clamp. 2) A single vertical incision was made at the site of the chalazion, the contents of the chalazion were removed completely, and the chalazion’s capsule was incised and removed. 3) The eye was bandaged with an eye patch after ofloxacin eye ointment had been applied. The patient was instructed to remove the patch 3 h later. Eye drops and eye ointment were used according to the methods described above.

All operations were performed by the same doctor in the outpatient procedure room on the same day of the examinations. All patients were followed up weekly before complete chalazion resolution.

### Statistical analyses

A paired-sample t test was used to compare meibomian gland function scores before and after complete chalazion resolution. The Wilcoxon rank test was used to compare the proportion of meibomian gland loss at 1 month after complete chalazion resolution and the proportion of the initial chalazion area. A *P* value less than 0.05 was considered statistically significant. The statistical software used was IBM SPSS Statistics Desktop Version 18.0 (IBM Corp, Armonk, NY).

## Results

### Basic patient information

Seventy-one (82 eyelids) patients were initially enrolled. At 1 month after complete chalazion resolution, 5 patients who received conservative treatment and 8 patients who underwent surgery were lost to follow-up. Finally, a total of 58 patients (67 eyelids), including 23 patients (23 eyelids) who received conservative treatment and 35 patients (44 eyelids) who underwent surgery, were included in the statistical analysis. Compared to those in patients who underwent surgery, the average chalazion size was smaller and the average chalazion duration was shorter in patients who received conservative treatment, but the average chalazion resolution time was longer. The basic information of the patients who received the two treatment methods is shown in Table 1.

**Table 1:** Baseline demographic and chalazion characteristics with conservative treatment and surgery

	Conservative treatment	Surgery
Number of patients	23	35
Number of eyelids	23	44
Age,mean ± SD,yr	32.39 ± 13.43 (14,57)	32.86 ± 14.64 (10,66)
Gender		
Male,n(%)	8 (35%)	14 (40%)
Female,n(%)	15 (65%)	21 (60%)
Chalazion location,n(%)		
RUL	10 (43%)	11 (25%)
RLL	5 (22%)	8 (18%)
LUL	5 (22%)	12 (27%)
LLL	3 (13%)	13 (30%)
Pre-treatment duration,mean ± SD,month	1.09 ± 0.87 (0.25,3)	5.93 ± 12.72 (0.25,24)
Chalazion size,mean ± SD,mm	5.22 ± 1.59 (2,8)	7.00 ± 1.77 (4,11)
Resolution time,mean ± SD,week	4.39 ± 1.00 (3,6.5)	1.98 ± 0.73 (1,4)

*Abbreviations: RUL* right upper eyelid, *RLL* right lower eyelid, *LUL* left upper eyelid, *LLL* left lower eyelid

### Changes in meibomian gland function in the chalazion area before and after complete chalazion resolution

Before treatment, meibomian orifice plugging was observed in the chalazion area in some patients, and meibum with a more opaque and toothpaste-like appearance was difficult to express. Meibomian gland function at 1 month after complete chalazion resolution was better than that before conservative treatment (*P* < 0.05). Meibomian gland function before surgery did not significantly differ from that at 1 month after complete chalazion resolution (*P* > 0.05) (Table 2).

**Table 2:** Changes of meibomian gland function score in the chalazion area before and after chalazion complete resolution

Mebomian gland function index		0 m Mean ± SD	1 m Mean ± SD	*t*	*P* value
Conservative treatment	Meibomian gland orifice	0.74 ± 0.75 (0,2)	0.35 ± 0.49 (0,1)	2.598	0.016
	Expressibility	0.48 ± 0.67 (0,2)	0.17 ± 0.49 (0,2)	2.612	0.016
	Meibum quality	1.22 ± 0.60 (1,3)	0.91 ± 0.60 (0,2)	3.102	0.005
Surgery	Meibomian gland orifice	0.93 ± 0.87 (0,2)	0.93 ± 0.82 (0,2)	0.025	0.980
	Expressibility	1.07 ± 0.70 (0,2)	0.95 ± 0.75 (0,2)	1.702	0.096
	Meibum quality	1.59 ± 0.76 (1,3)	1.52 ± 0.70 (1,3)	1.774	0.083

*0 m* before treatment, *1 m* 1 month after chalazion complete resolution

### Changes in meibomian gland morphology in the chalazion area

**Fig. 2:**
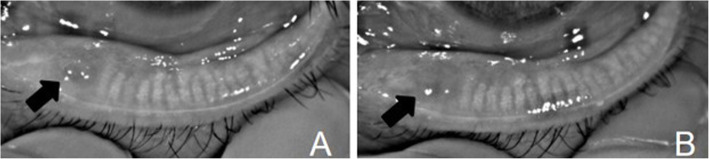
The meibography images of chalazion with conservative treatment. **a** shows the chalazion of meibography image before treatment; **b** shows the meibomian gland loss at 1 months after chalazion resolution

**Fig. 3:**
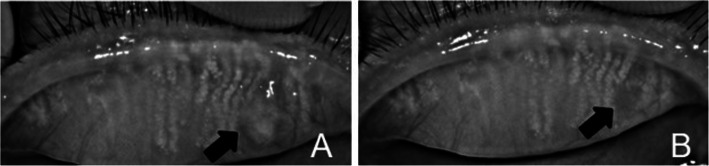
The meibography images of chalazion with surgery. **a** shows the chalazion of meibography image before surgery; **b** shows the meibomian gland loss at 1 months after chalazion resolution

Normal meibomian gland morphology in the chalazion area could not be observed before treatment, but after complete chalazion resolution, meibomian gland loss was observed in the chalazion area with both treatment methods. Moreover, no statistically significant difference between the proportion of meibomian gland loss and the proportion of the initial chalazion area (*P* > 0.05) was noted in either treatment group (Table 3) (Figs. 2 and 3).

**Table 3:** Comparison of the proportion of meibomian gland loss after chalazion complete resolution and the proportion of initial chalazion area

Area proportion(%)	0 m M(P_25_,P_75_)	1 m M(P_25_,P_75_)	Z	*P* value
Conservative treatment	14.90 (11.03,25.3)	14.64 (10.33,25.77)	−1.171	0.242
Surgery	14.90 (12.04,21.6)	14.84 (11.31,21.81)	−0.070	0.944

*0 m* before treatment, *1 m* 1 month after chalazion complete resolution

### Changes in the acinar structure in the chalazion area

**Fig. 4:**
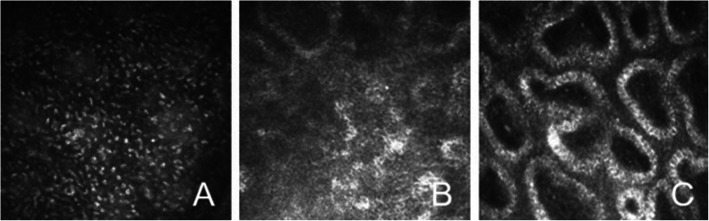
In vivo confocal microscopy images in chalazion area before and after treatment. **a** shows a large number of inflammatory cells in the chalazion area before treatment; **b** shows incomplete acinar structure in the range of meibomian gland loss at 1 months after chalazion resolution. **c** shows normal acinar structure of meibomian gland

In this study, we intended to observe the acinar structure in the chalazion area at the cellular level. However, due to the limitation of current ophthalmic in vivo LSCM [13, 14], clear acinar images can be obtained only at or near the eyelid margin. In our study, only 3 patients (3 eyelids) with conservative treatment had chalazia located near the eyelid margin, and in vivo LSCM examination revealed a large number of inflammatory cells in the chalazion area before treatment. At 1 month after complete chalazion resolution, no obvious inflammatory cells were present, but the intact acinar structure was not found at the area. (Fig. 4).

## Discussion

The findings from this non-randomized, prospective observational clinical study demonstrate that chalazion causes meibomian gland loss, and the range of meibomian gland loss after complete chalazion resolution is related to the range of chalazion itself, that is, the range of chronic granulomatous inflammation. Interestingly, despite our initial concern, surgery did not expand the range of meibomian gland loss. One previous study reported that chalazion itself or chalazion excision may cause meibomian gland loss; however, due to the limitations of the retrospective study design, the factor responsible for meibomian gland loss was unclear [15]. This finding clearly supplemented the conclusions of the study. According to monitoring of the progression of chalazia by infrared meibomian gland photography, we found that the meibomian gland morphology could not be observed in the chalazion area before treatment (conservative treatment or surgery), and after complete chalazion resolution, the area showed meibomian gland loss. In this study, the calculation method of the proportion of the chalazion area was the chalazion area/the whole area of the tarsal plate, although the lower eyelid was everted more difficult than the upper eyelid, The calculation method of the whole area of the tarsal plate (original meibomian gland area) was the same as upper eyelid, that is, total area of tarsal plate can be seen in the image. The total area of the tarsal plate was only measured once (before treatment), so, in this formula the denominator remains unchanged before and after treatment. As the results show, no statistically significant difference was found between the range of meibomian gland loss after complete chalazion resolution and the range of the initial chalazion area before treatment using either method. The reasons for these results are discussed below.

Chalazion formation is a typical physiological response to chronic granulomatous diseases [1]. Previous studies have shown that chalazia mainly involves macrophages, neutrophils and epithelial cells [16, 17]. Moreover, the lipid component of chalazion differs from normal meibomian gland lipids [17]. However, infrared meibomian gland photography is based on the theory that the lipid particles in a meibomian gland are excited by infrared rays and emit scattered light [18]. A chalazion is composed of granulomatous substances and abnormal lipids before treatment. Therefore, the meibomian gland morphology cannot be observed in the chalazion area.

A previous study reported that when inflammatory cells infiltrated the meibomian glands, normal meibum secretion was blocked, but abnormal meibum continued to be produced, and these changes led to increased pressure in the glands, which may cause meibomian gland structure destruction [19]. Accordingly, the chalazion area showed meibomian gland loss after complete resolution.

The normal tarsal plate is composed of a dense connective tissue plate, and meibomian glands are embedded in the tarsal plate of the eyelid [9], with no cystic cavity formation. Because of the local granulomatous reaction, a chalazion (cystic cavity) forms on the tarsal plate, which is an inflammatory lesion. The range of incision and curettage is the range of the cystic cavity. Therefore, regardless of whether conservative treatment or surgery is applied, self-absorption or curettage involves only the granuloma reaction and does not involve the normal tarsal tissue. Therefore, the range of meibomian gland loss is related to the range of chalazion itself rather than the treatment method. One previous study also compared infrared meibography before and after curettage for the chalazion. As a result, when comparing meibography of the baseline with meibography at 1 month after incision and curettage of the chalazion, the normal area of the meibomian gland in the chalazion significantly increased (*p* = 0.041). But, they didn’t think that the acini regenerated after resolution of the chalazion [20].

A previous study found that the ratio of cholesterol/cholesterol esters (Chl/CE) was increased in the abnormal lipid component of chalazion and suggested that an increase in Chl levels might send a chemotactic signal to inflammatory cells to invade the meibomian gland [17]. The inflammatory cells may cause a physical blockage of the meibomian gland or meibum thickening, which would obstruct the expression of meibum. As a result, this abnormal lipid material might invade the surrounding tissues and intensify the inflammatory response in the meibomian glands. This vicious cycle may exist during the formation and expansion of a chalazion before treatment, and occasionally, a chalazion may enlarge even during conservative treatment. As mentioned above, the range of meibomian gland loss was related to the range of chalazion itself. Therefore, the best strategy to reduce the range of meibomian gland loss is to cure the chalazion as soon as possible. Based on our observation, chalazion can be cured rapidly by surgery. In fact, one study showed that complete resolution rates were low for three conservative treatment methods and suggested that ophthalmologists, particularly those in subspecialty clinics such as oculoplastics, can use surgical or invasive therapy earlier during treatment [6]. Another study showed that incision and curettage was a good therapeutic choice for all chalazia [8]. Despite such recommendations, many ophthalmologists choose to try conservative treatment first even if the chalazion size is large and the chalazion duration is long because they fear that surgery will damage the meibomian glands. However, our result showed that surgery did not expand the range of meibomian gland loss. In addition, a previous study considered that starting with surgical options earlier may also reduce patients’ exposure to antibiotics and/or steroids, which may cause antibiotic resistance or increased intraocular pressure and steroid-induced glaucoma when overused. Therefore, we suggest that ophthalmologists perform surgery sooner during treatment to cure chalazion rapidly and thus control the range of meibomian gland loss in a short time.

In this study, we found meibomian orifice plugging and toothpaste-like meibum in one or several meibomian glands where chalazion was located before treatment, which may suggest that local meibomian gland function had already changed before chalazion formed, and meibomian gland obstruction may lead to chalazion, which was also one of the causes of chalazion formation [9]. As the results showed, meibomian gland function improved at 1 month after complete chalazion resolution with conservative treatment. However, no statistically significant differences were noted before and after surgery. One reason was patients with conservative treatment used hot masks before complete chalazion resolution, and frequent and regular heating melted the meibum of the non-chalazion part, allowing the meibum to discharge on the eyelid margin [21–23]. Another reason was the improvement of meibomian gland function might differ between the two groups, because the size of chalazion was smaller in the conservative group, but chalazion size is often related to the pre-treatment duration in the clinic, not meibomian gland function. Moreover, hot compress is an effective method to improve meibomian gland function [22], in this study, hot compresses was only used in conservative group. So we consider the improvement of meibomian gland was related to hot compress.

Notably, complete chalazion resolution does not indicate the end of treatment. If meibomian gland function in the non-chalazion area does not improve, the meibomian glands will become obstructed and cause chalazion again, which is why some people are prone to suffer from chalazion. No specific preventive strategy is available for chalazia because chalazion is closely related to the meibomian gland. Previous studies have shown that meibomian gland function can be improved by various methods. A hot compress combined with meibomian gland massage is considered a traditional and effective method [6, 22], while LipiFlow treatment and intense pulsed light (IPL) treatment are optional choices [24, 25], and intraductal meibomian gland probing can be used in patients with severe meibomian gland obstruction [26]. Therefore, further research on personalized treatments according to the condition of meibomian glands after complete chalazion resolution is needed.

In this study, only 3 patients had a chalazion located near the eyelid margin, and the intact acinar structure was not found at the area after complete chalazion resolution. The reason for this result may be that the meibomian gland is a special type of sebaceous gland with a holocrine acinar secretion pattern, indicating that the contents of the whole glandular cells form the meibum. Normally, the basal layer of meibocytes in the periphery of the acinus contains a proliferating progenitor cell population that constantly gives rise to new meibocytes, and the above process is repeated [3]. However, when inflammatory cells infiltrate the gland, new meibocytes cannot be formed in the short term; therefore, the complete acinar structure was not observed in the chalazion area. However, this result does not indicate that new meibocytes will never regenerate. A previous study reported stem cells of the meibomian glands located at the circumference of each acinus, which were responsible for the continuous generation of meibocytes; approximately 13 days is required for newly formed meibocytes to eventually shed in the mouse meibomian gland [27]. Perhaps due to the effects of inflammation, new meibocytes need more time to regenerate. Moreover, another study showed that the meibomian gland can regenerate with meibomian gland probing [28]. Therefore, whether meibocytes can regenerate and the regeneration time after chalazion resolution require further study.

Our study has some limitations. The dry eye tests, such as Schirmer 1, T-BUT, ocular surface staining score, etc. were not be observated, we thus were not able to observe the effect of chalazion on the ocular surface. This will be the direction of our future research. In this study, patients could not be randomly divided into conservative treatment or surgery groups because such stratification was not possible given the actual clinical situation. The treatment decision depends on the chalazion size, the lesion duration, the patient’s consent and so on. In addition, the number of cases observed by confocal microscopy was small and not representative. To determine whether the acinar also disappears in the meibomian gland loss area, a larger sample size is needed.

## Conclusion

In summary, chalazion as a form of chronic granulomatous inflammation causes meibomian gland loss, and the range of meibomian gland loss after chalazion resolution is not related to the treatment method but to the range of the chalazion itself. Given that surgery does not aggravate meibomian gland loss but rather facilitates faster complete chalazion resolution, we suggest that ophthalmologists, particularly those in subspecialty clinics, can perform surgery earlier. At the same time, complete chalazion resolution should not be the ultimate goal of treatment as improving meibomian gland function in the non-chalazion area is more important.

## Data Availability

The datasets used and/or analyzed during the current study are available from the corresponding author on reasonable request.

## Abbreviations

LSCM: Laser scanning confocal microscopy
MGD: Meibomian gland dysfunction
BKC: Blepharokeratoconjunctivitis
IPL: Intense pulsed light
